# Natural killer cell activity for IFN-gamma production as a supportive diagnostic marker for gastric cancer

**DOI:** 10.18632/oncotarget.19712

**Published:** 2017-07-31

**Authors:** Jongmi Lee, Ki Hyun Park, Ji Hyeong Ryu, Hyun Jin Bae, Aeran Choi, Hyeyoung Lee, Jihyang Lim, Kyungja Han, Cho Hyun Park, Eun Sun Jung, Eun-Jee Oh

**Affiliations:** ^1^ Department of Laboratory Medicine, Seoul St. Mary’s Hospital, College of Medicine, The Catholic University of Korea, Seoul, Korea; ^2^ Department of Biomedical Science, Graduate School, The Catholic University of Korea, Seoul, Korea; ^3^ SamKwang Medical Laboratories, Seoul, Korea; ^4^ Division of Gastrointestinal Surgery, Department of Surgery, Seoul St. Mary’s Hospital, College of Medicine, The Catholic University of Korea, Seoul, Korea; ^5^ Department of Hospital Pathology, Seoul St. Mary’s Hospital, College of Medicine, The Catholic University of Korea, Seoul, Korea

**Keywords:** natural killer cell activity, interferon-gamma, diagnostic marker, gastric cancer

## Abstract

**Background/Aim:**

Decreased Natural killer cell activity (NKA) for interferon-gamma production (NKA-IFNγ) has been reported in cancer patients. The aim of this study was to determine the diagnostic performance of NKA-IFNγ for gastric cancer (GC).

**Results:**

NKA-IFNγ levels were decreased in 261 GC patients with all stages of tumor compared to those in 48 healthy donors (*P <* 0.001), and lower levels of NKA-IFNγ were associated with higher GC stages. NKA-IFNγ levels were also associated with clinicopathological parameters including tumor size, depth of invasion, and lymph node metastasis. NKA-INFγ assay had better diagnostic value (AUC = 0.822) compared to serum CEA (0.624) or CA19-9 assay (0.566) (*P* < 0.001). Using different cut-off levels, serum CEA and CA19-9 showed sensitivities of 6.1-14.2% and 4.2-28.0%, respectively, which were much lower than that of NKA-IFNγ (55.6-66.7%).

**Methods:**

This study included 261 patients with newly diagnosed GC and 48 healthy donors. NKA for IFNγ was determined by enzyme immunoassay after incubation of whole blood, and diagnostic performance was evaluated.

**Conclusions:**

NK cell activities for IFNγ production could be used as a supportive non-invasive tumor marker for GC diagnosis.

## INTRODUCTION

Gastric cancer (GC) is one of the most prevalent cancers. It remains a major global public health problem [1]. Although early detection of GC and metastasis is crucial to achieve better outcome for GC, conventional non-invasive serum tumor markers such as carcinoembryonic antigen (CEA), cancer antigen 125 (CA125), and cancer antigen 19–9 (CA19-9) have poor diagnostic values for GC mainly due to their low sensitivities [2].

Natural killer (NK) cells play a vital role in immune response to cancer by identifying and killing cancer cells with reduced or absent MHC-class I expression or by expressing stress-induced molecules [3, 4]. They have direct cytotoxicity through perforin and granzyme release, and they also control immune response by secreting cytokines such as interferon-gamma (IFNγ) and tumor-necrosis factor-α [4]. In previous study, NKA is stable in individuals, and the level of NKA remains unaffected over years unless events occurs [5]. However, the activity of NK cells shows heterogeneity among different persons, which may induce tumor development and decreased INFγ production has been observed since early stage of malignancy [6]. In cancer patients, peripheral NK cells revealed reduced cytotoxic function [7–10], and significantly decreased intracellular IFNγ productions were reported [4, 11]. Therefore, measuring NKA for IFNγ secretion (NKA-IFNγ) might be valuable for screening GC and estimating cancer stage. However, reports on the diagnostic value of NKA levels in cancer patients are limited due to variability and inconsistency in measurement of NKA levels.

NK Vue-Kit (ATgen, Sungnam, Korea) is an *in vitro* diagnostic test system that can measure NKA-IFNγ levels using sandwich enzyme immunoassay. The principle of NK Vue test is to stimulate whole blood with engineered recombinant cytokines that specifically activates NK cells and to measure the levels of IFNγ cytokine released from activated NK cells. Previous studies have reported that patients with malignancy have significantly decreased levels of NKA-IFNγ compared to healthy subjects [8, 9], suggesting that NKA-IFNγ might be useful as a supportive diagnostic marker of malignancy. However, the diagnostic value of NKA-IFNγ in GC patients has not been evaluated yet. Little is known about the association between NKA-IFNγ levels and clinicopathological parameters or current laboratory tests.

Therefore, the objective of the present study was to compare the diagnostic performances of NKA-IFNγ to other tumor markers (serum CEA and CA19-9) in GC patients and examine the correlation between tumor markers and clinicopathologic parameters.

## RESULTS

### Baseline characteristics of patients

Demographics of the study population are shown in Table 1. The healthy donor group had 23 males and 25 females with a mean age of 43.5 ± 10.4 years. Of 261 patients with newly diagnosed GC, 157 (60.2%) were at stage I, 35 (13.4%) were at stage II, 35 (13.4%) were at stage III, and 34 (13.0%) were at stage IV.

**Table 1:** Demographic characteristics of healthy donors and gastric cancer patients used in this study

	Healthy donors (n=48)	GC patients (n=261)	*P*-value
Age, years	43.5 ± 10.4^*^	60.5 ± 12.5	< 0.001
Male, N (%)	23 (47.9)	162 (62.1)	0.066
WBC, N per mL	5.6 ± 1.6^*^	6.5 ± 2.4	0.004
Lymphocyte, %	34.8 ± 8.7^*^	31.7 ± 9.5	0.024
Lymphocyte, N per mL	1891.1 ± 649.4^*^	1973.0 ± 639.9	NS
NKA-IFNγ, pg/mL	1520 (818.6-1970.1)^†^	204.8 (174.4-260.5)	< 0.001
CEA, ng/mL	0.8 (0.6-1.4)^†^	1.4 (1.3-1.6)	0.010
CA19-9, U/mL	6.6 (4.6-9.6)^†^	8.8 (7.4-9.7)	NS
Tumor stage, N(%)			
I		157 (60.2)	
II		35 (13.4)	
III		35 (13.4)	
IV		34 (13.0)	

*Mean ± SD, †median (95% CI), N= number, NS= not significant.

### NKA-IFNγ, serum CEA, and CA19-9 levels in healthy donors and GC patients

**Figure 1:**
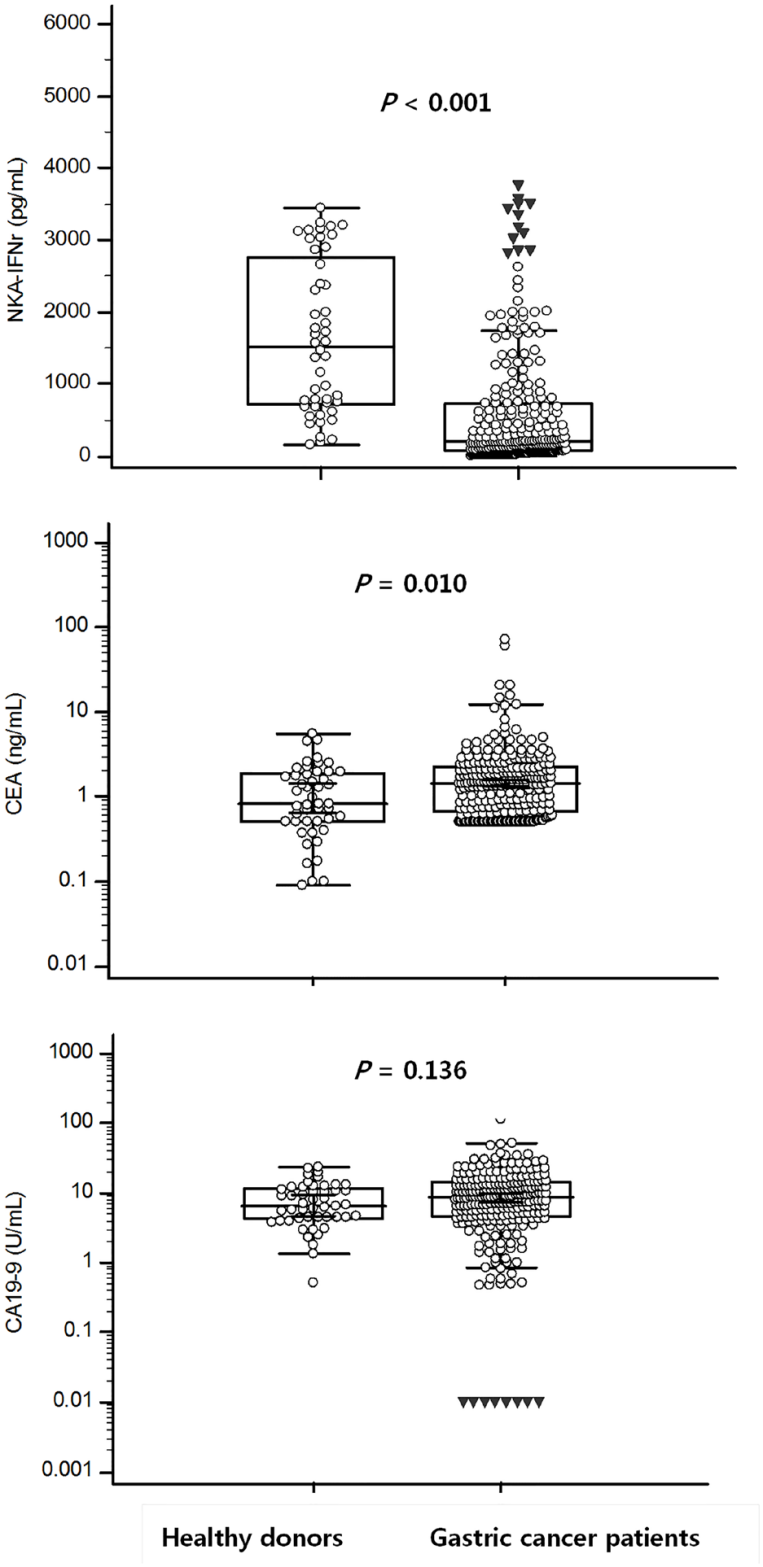
NKA-IFNγ, CEA, and CA19-9 levels from healthy donors compared with that from gastric cancer patients In box plots, central box represent the 25th to 75th centiles, the lines within the boxes represent the median and error bars represent 95% confidence interval for the medians.

**Figure 2:**
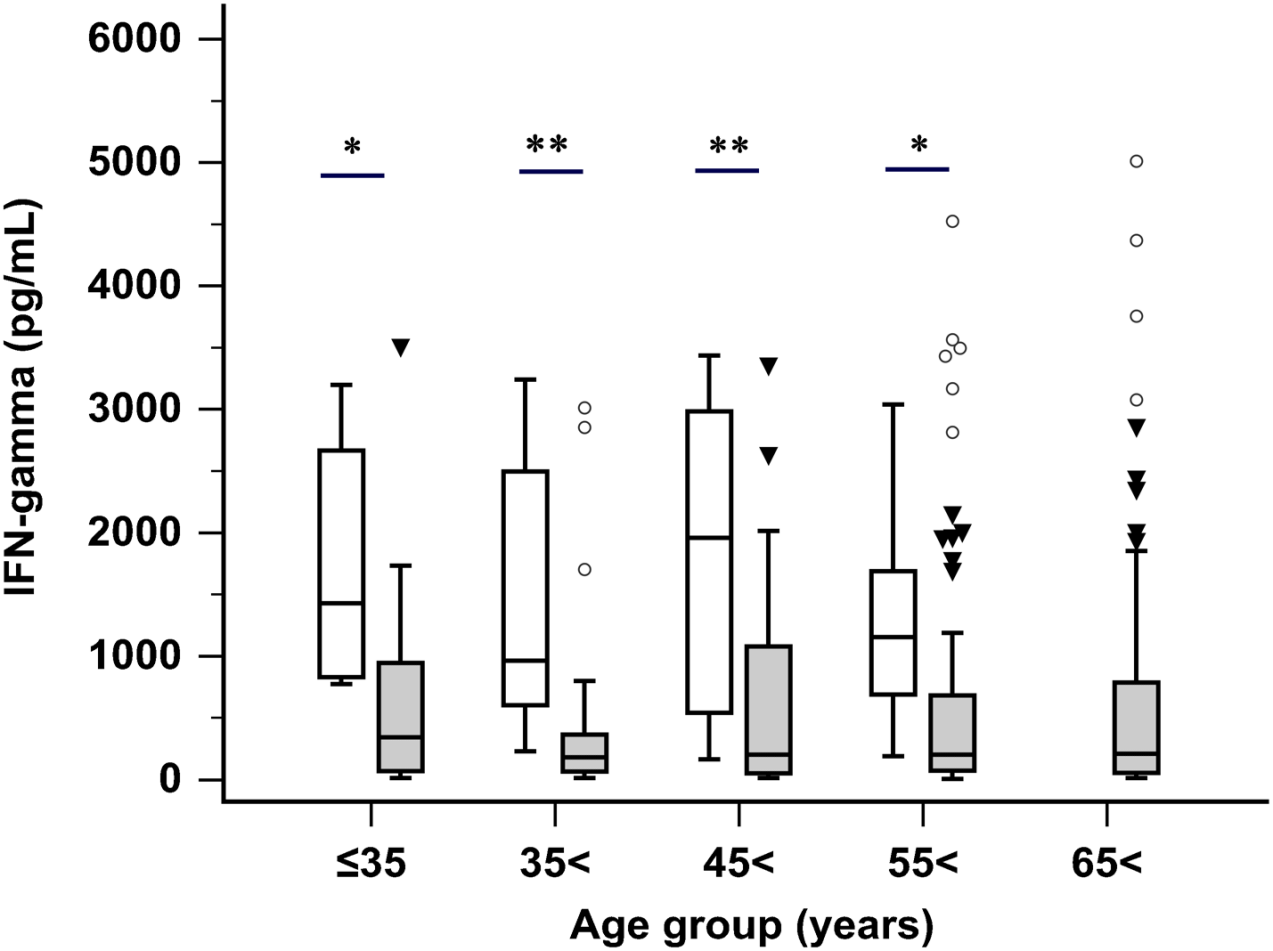
NKA-IFNγ levels in healthy donors (white boxes) compared to those in gastric cancer patients (gray boxes) in different age groups (groups; ≤ 35 years, 36-45 years, 46-55 years, 56-65 years and 65< years) (*P < 0.05; ** *P* < 0.001). In box plots, central box represent the 25th to 75th centiles, the lines within the boxes represent the median and error bars represent 95% confidence interval for the medians.

Levels of NKA-IFNγ, serum CEA, and serum CA19-9 in healthy donors and GC patients are shown in Figure 1. GC patients had significantly lower NKA-IFNγ levels compared to healthy donors [median (95% CI): 204.8 (174.4 to 260.5) pg/mL vs. 1,520 (818.6-1970.1) pg/mL, *P <* 0.001]. NKA-IFNγ levels in 48 healthy donors ranged from 160.7 to 3435.0 pg/mL. There was no significant correlation between age and NKA-IFNγ levels in healthy donors (r = -0.130, *P* = 0.379) and in GC patients (r = -0.033, *P* = 0.601). Comparison of NKA-IFNγ levels between healthy donors and GC patients showed that NKA-IFNγ levels were decreased in all age groups of GC patients (≤ 35 years, *P* = 0.022; 36-45 years, *P* < 0.001; 46-55 years, *P* < 0.001; 56-65 years, *P* = 0.028) (Figure 2). Serum CEA concentrations in GC patients were significantly (*P =* 0.010) higher than those of healthy donors (Figure 1). Of 261 GC patients, 15 (5.7%) patients were positive for serum CEA when upper limit of reference range at 5.0 ng/mL was used according to the manufacturer. Serum CA19-9 levels were not significantly different between GC patients and healthy donors [8.8 (7.4 to 9.7) U/mL vs. 6.6 (4.6 to 9.6) U/mL, *P =* 0.136]. Of 261 GC patients, 11 (4.2%) were positive for serum CA19-9 when cut-off value of 36.1 U/mL was used according to the manufacturer’s instruction.

### Tumor marker levels in GC patients according to TNM stage

**Figure 3:**
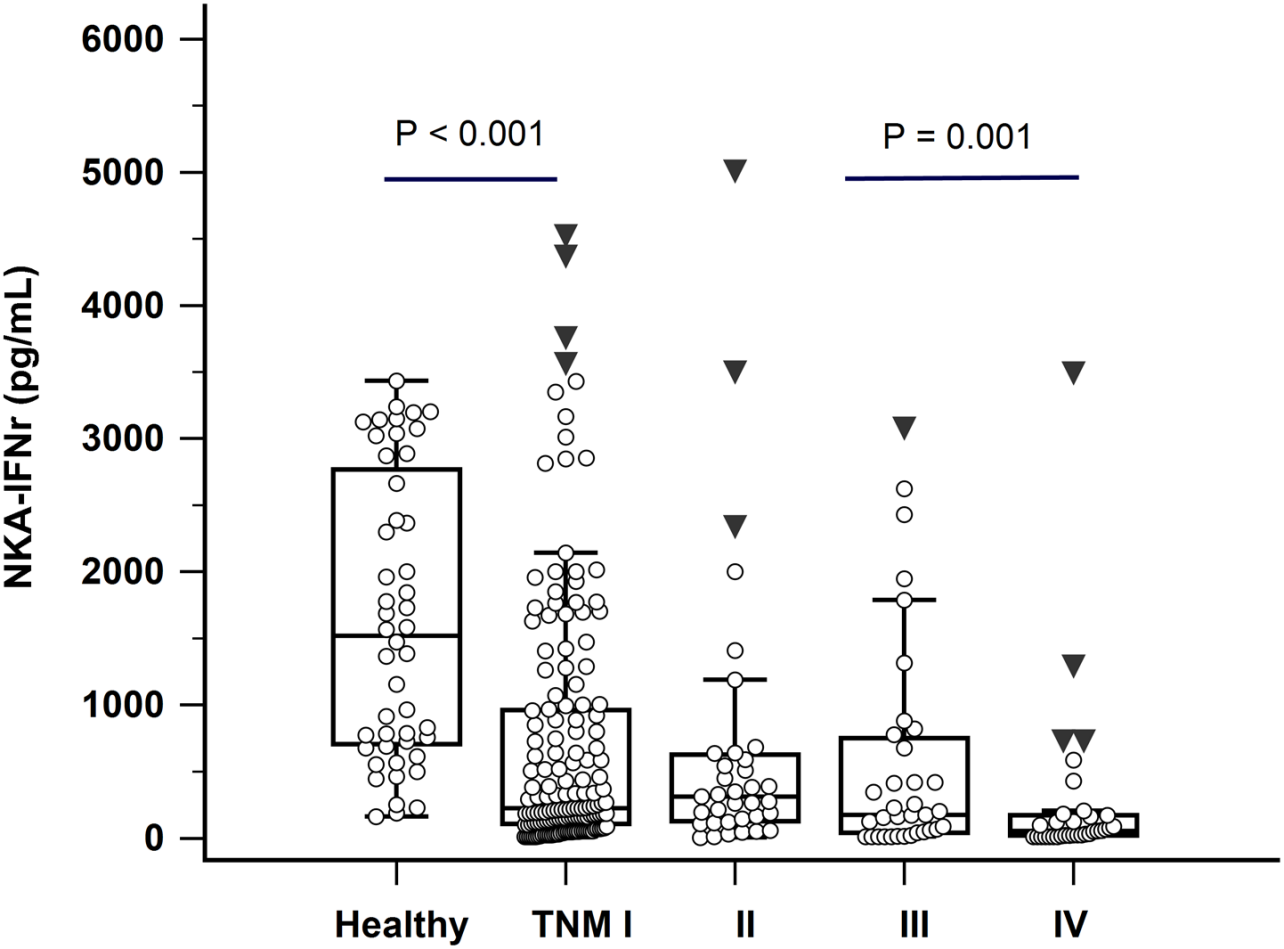
NKA-IFNγ levels in healthy donors and gastric cancer patients according to TNM stage (I-IV) In box plots, central box represent the 25th to 75th centiles, the lines within the boxes represent the median and error bars represent 95% confidence interval for the medians.

NKA-IFNγ levels were decreased in patients with advanced TNM stages (Figure 3). GC patients with distant metastasis (TNM stage IV) had significantly lower NKA-IFNγ levels than GC patients with stage II or III (*P =* 0.001). Interestingly, NKA-IFNγ levels in GC patients with TNM stage I were also significantly lower than those of healthy donors (*P <* 0.001). In terms of serum CEA and CA19-9 levels, serum CA19-9 levels were only significantly higher in patients with distant metastasis (TNM stage IV) compared to those in patients without metastasis (TNM stage I, II, or III) (*P =* 0.006). Serum CEA levels were not significantly different among GC patients with different TNM stages (*P* > 0.05).

### Association of tumor marker levels with pathologic parameters in GC patients

Results of association of NKA-IFNγ, serum CEA, and CA19-9 levels with pathologic parameters are shown in Table 2. Tumor size of ≥ 5 cm only showed correlation with NKA-IFNγ levels among three tumor markers. Depth of invasion (T3, T4), lymph node invasion, and distant metastasis had significant association with both NKA-IFNγ and serum CA19-9 levels (*P <* 0.05). However, serum CEA levels showed no correlation with any pathologic parameters (tumor size, histologic grade, depth of invasion, lymph node metastasis, or distant metastasis).

**Table 2:** NKA-IFNγ, CEA, and CA19-9 levels of in 261 gastric cancer patients according to pathologic parameters

	N	NKA-IFNγ(pg/mL)	*P*-value	CEA (ng/mL)	*P*-value	CA19-9 (U/mL)	*P*-value
Tumor size (cm)							
<5	174	217.2 (183.6 - 310.4)	0.019	1.4 (1.2-1.5)	NS	8.1 (6.6-9.1)	NS
≥5	87	174.0 (66.9-276.6)		1.5 (1.3-2.0)		10.3 (8.8-13.4)	
Histologic grade							
Well to moderate	81	207.0 (160.0-260.3)	NS	1.7 (1.5-1.9)	NS	8.5 (6.2-10.1)	NS
Poor and others	180	202.2 (172.2-310.6)		1.3 (1.0-1.4)		8.9 (7.2-10.2)	
Depth of invasion							
T1, T2	168	226.0 (191.8-323.1)	0.004	1.4 (1.2-1.6)	NS	7.2 (6.2-8.7)	<0.001
T3, T4	93	162.0 (88.0-226.4)		1.5 (1.3-1.8)		11.2 (9.7-14.2)	
Lymph node invasion							
absent	146	278.1 (204.2-431.2)	<0.001	1.4 (1.2-1.6)	NS	8.2 (6.7-9.4)	NS
present	103	162.0 (108.8-224.2)		1.5 (1.3-1.9)		9.6 (7.7-10.3)	
Distant metastasis							
absent	228	240.5 (194.7-329.4)	<0.001	1.4 (1.3-1.5)	NS	8.5 (7.1-9.3)	0.007
present	33	53.0 (23.2-109.5)		1.7 (1.1-2.5)		13.6 (8.3-25.2)	

N, number of GC patients; Data are represented as median (95% CI); NS, not significant.

### Diagnostic performances of three tumor markers using normal and optimum cut-off values

**Figure 4:**
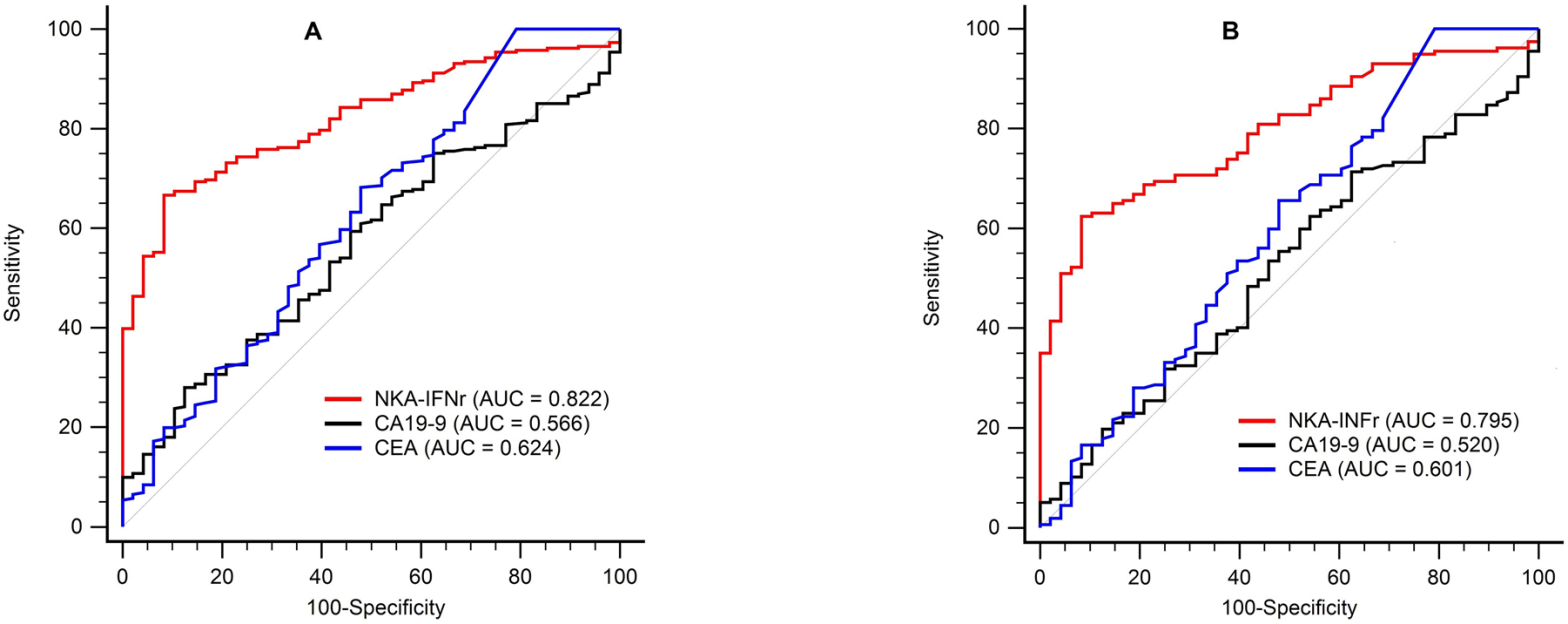
ROC curves analysis of NKA-IFNγ, CEA, and CA19-9 assays to discriminate healthy donors from gastric cancer (GC) patients (A) and early GC patients (B)

The ability of tumor marker levels to diagnosis of GC patients was evaluated by ROC curve analysis. AUCs of NKA-IFNγ, CEA, and CA19-9 were 0.822, 0.624, and 0.566, respectively (Figure 4). The AUC value for NKA-IFNγ was significantly higher than that for CEA or CA19-9 (both *P <* 0.001). Using the normal cut-off provided by the manufacturer, three tumor markers had good specificity (91.7 -100.0%) but poor sensitivity (4.2 - 55.6%) for the detection of GC patients (Table 3). Diagnostic values were determined using optimum cut-off values determined by ROC curve. When NKA-IFNγ level was less than 438 pg/mL, NKA-IFNγ showed the best sensitivity (66.7%) with specificity (91.7%) and an AUC of 0.822. Sensitivities of serum CEA and CA19-9 using optimum cut-off values were improved to 14.2% and 28.0%, respectively. However, they were still too low for screening GC patients. The NKA-IFNγ levels were not correlated to the CEA (r = 0.114) or CA-19-9 levels (r = -0.088). However, only NKA-IFNγ was independent variable according to multivariate logistic stepwise regression analysis (*P* < 0.001). The combination of NKA-IFNγ, CEA and CA19-9 for gastric cancer improved sensitivity (78.9%), but specificity dropped to 68.8% (Table 3). When we analyzed the diagnostic performance for early GC detection, the AUCs values of NKA-IFNγ, CEA, and CA19-9 were 0.795, 0.601, and 0.520, respectively (Figure 4B). Using the optimum cut-off values (438 pg/mL), the sensitivity and specificity of NKA-IFNγ for early GC were 62.4% (54.3-70.0) and 91.7% (80.0-97.7), respectively.

**Table 3:** Diagnostic accuracies of studied tumor markers for gastric cancer

	AUC(95% CI)	Cut-off	Sensitivity(95% CI)	Specificity(95% CI)
NKA-IFNγ, pg/mL	0.822 (0.774-0.863)	250.0^*^	55.6 (49.3-61.7)	91.7 (80.0-97.7)
		438.0^†^	66.7 (60.6-72.4)	91.7 (80.0-97.7)
CEA, ng/mL	0.624 (0.567-0.678)	5.0^*^	6.1 (3.5-9.8)	97.9 (88.7-99.9)
		3.0^†^	14.2 (10.2-19.0)	93.8 (82.5-98.7)
CA19-9, U/mL	0.566 (0.509-0.622)	36.1^*^	4.2 (2.1-7.4)	100.0 (92.6-100.0)
		13.3^†^	28.0 (22.7-34.0)	87.5 (74.8-95.3)
‡NKA-IFNγ + CEA			71.7 (65.8-77.0)	85.4 (72.2-93.9)
‡NKA-IFNγ + CA19-9			76.6 (71.0-81.6)	75.0 (60.4-86.4)
‡NKA-IFNγ + CEA+ CA19-9			78.9 (73.5-83.7)	68.8 (53.8-81.3)

*normal cut-off levels provided by manufacturers, †optimum cut-off levels by ROC curve analysis, ‡combination of tumor markers using optimum cut-off levels.

## DISCUSSION

In present study, the diagnostic value of NKA-IFNγ for gastric cancer was compared to another two serum markers (CEA, CA19-9). When NKA-INFγ levels in GC patients and healthy donors were compared, GC patients had significantly lower NKA-INFγ levels. In clinical laboratory, several methods have been introduced to measure NK function in different diseases [12–14]. However, methods for determining NK activities are different depending on study groups, and they have not been standardized yet. Radioactive chromium (^51^Cr) release assay has been considered as the gold standard method for measuring NK activity. However, it has several limits, such as radioactivity, inter-laboratory variability, and low sensitivity for apoptotic cell death [12]. Although alternative method such as flow cytometry based NK cytotoxicity assay has been used, this test requires large amount of whole blood for PBMC or isolated NK cell. In addition, experimental processes involved in such assay are labor intensive. In the present study, we measured INFγ production to determine NKA. A previous study has demonstrated that IFNγ cytokines measured by NKvue kit are mainly released from NK cells and that secreted IFNγ levels typically represent NK cell activation and effector function [8]. In this study, NK cells in whole blood were immediately (less than 1 hour) activated by mixing with PromocaH stimuli without delay for sample preparation and soluble IFNγ levels in culture supernatants were measured by ELISA.

In terms of reference values for NKA-IFNγ assay, the manufacturer has provided normal cut-off value as > 250 pg/mL. Previous studies have been reported reference values of 867.5 ± 50.2 (mean ± SD) pg/mL [8] and 975.2 ± 85.7 pg/mL [9]. In the present study, we analyzed NKA-IFNγ levels in 48 healthy donors for organ transplantation. Their NKA-IFNγ levels were at 1,520 (818.6 - 1970.1) [median (95% CI)] pg/mL. Frequency distribution analysis in present study showed that NK activities were not normally distributed, and they did not display equal variance. Therefore, we could not absolutely compare NK activities between age matched healthy donors and GC patients. However, significant difference in IFNγ levels was not found according to gender or age in healthy donors or gastric cancer patients, consistent with results of previous report [8].

Although defect of NK cell activity were previously reported in patients with several cancers including melanoma, lung, and breast cancers [15–17], NK cell activities were only decreased in advanced metastatic patients. In present study, NKA-IFNγ levels were decreased in different age groups of GC patients. In addition, GC patients with all stages of tumor, including those with stage I gastric cancer had lower NKA-IFNγ levels compared to those from healthy donors. This finding suggests that NK cells are dysregulated in cancer patients from early stages of GC, and defect of NK cell function may be a substrate for tumor development. Therefore, measurement of NK function might be useful for GC screening.

Previous studies have revealed that circulating NK cells might present a role in metastasis’s control as mice with NK cell depletion have shown significantly increased overall metastatic activity [6, 18–20]. However, the relationship between NK cells and tumor stage is controversial [16, 17, 21, 22]. In present study, we demonstrated that NKA-INFγ levels were significantly lower in patients with higher TNM stages and NK cell activity was negatively correlated with GC stages. They were also significantly lower in patients with distant metastasis than those in patients without metastasis. In addition, decreased NKAs were associated with tumor size of more than 5 cm and lymph node metastasis in GC patients, confirming results of previous study in GC patients by NK cytotoxicity test [6]. Of note, all patients in our study did not receive chemotherapy or surgery. These findings confirmed that NK cells activation inhibit the metastasis in GC.

For screening GC, several non-invasive biomarkers (tumor markers) such as CEA, CA125, and CA19-9 have been widely used for the diagnosis of GC [23]. However, sensitivities of individual or combined tumor markers with different cut-off values are reported to be as low as 4.7 – 47.7% [2, 24–26]. In present study, serum CEA and CA19-9 showed sensitivities of 6.1-14.2% and 4.2-28.0%, respectively, using normal or optimum cut-off levels. These were much lower than the sensitivity of NKA-IFNγ (55.6-66.7%). ROC curve analysis also revealed that NKA-INFγ assay had better (*P* < 0.001) diagnostic value (AUC = 0.822) compared to serum CEA assay (AUC = 0.624) or CA19-9 assay (AUC = 0.566). Although CEA levels were significantly increased in GC patients compared to those in healthy donors (*P =* 0.01), majority of GC patients had levels of CEA within normal ranges. In multivariate regression analysis, only NKA-IFNγ was independent marker, and combination of NKA-IFNγ, CEA and CA19-9 for gastric cancer had 78.9% sensitivity and 68.8% specificity. When tumor marker levels between healthy donors and early GC patients (TNM stage I) were compared, only NKA-INFγ discriminated the two groups with 62.4% sensitivity and 91.7% specificity, indicating that further prospective studies should be conducted for healthy people with low NKA INFγ levels.

The mechanism of NKA result is not fully understood yet. In this study, we measured INFγ released from stimulated NK cells in 1 ml of fresh whole blood. NKA would be decreased simply due to NK cell inactivation or NK cell depletion. It has been suggested that such decreased NKA is not attributable to decreased number of NK cells, but due to impaired regulating system in cancer patients [20, 27–30]. In addition, decreased production of IFNγ due to unknown humoral inhibiting factors released from the tumor has been reported [31, 33]. Recently, Peng et al. showed that gastric cancer- associated secretion of TGFb1 from monocyte/macrophage induced the functional impairment of NK cells within gastric cancer tumors [32].

Potential limitations of present study include its relatively small number of normal healthy donors and its retrospective nature without evaluating prognostic impact or mechanism of NKA-IFNγ levels. It is also important to note that this study is focused on GC patients in comparison with healthy donors, not patients with benign gastric disease or other cancers. Regarding the NKA-IFNγ levels in benign gastric diseases, Lindgren et al. reported that NK cells from asymptomatic Helicobacter pylori (H. Pylori)-infected individuals had higher ability to produce IFNγ after stimulation compared to gastric cancer patients [34]. H. pylori infection is a major cause of chronic gastric inflammation, gastritis, peptic ulcer, involved in the development of gastric cancer [35]. When we additionally tested NKA-IFNγ in nine patients with benign gastric diseases - atropic gastritis (n=3), gastric polyp (n=4) and erosive gastritis (n=2)- but in whom H. pylori was not identified, the median (95%CI) NKA-IFNγ levels were increased to 1143.3 (332.9-1994.5) pg/mL. Because NK activities can be modulated by the different types of H. pylori antigens [36] and it tends to disappear when advanced atrophy and intestinal metaplasia extend [35], a prospective investigation of NKA-IFNγ in patients with benign gastric disease as well as patients with other cancers should be carried out to validate the diagnostic performance of NKA-IFNγ assay.

Despite these limitations, our results indicate the need for further longitudinal prospective studies to determine whether NKA-IFNγ assay can be used to detect GC patients early and predict their prognosis. Because NKA-IFNγ levels are also associated with the progression of cancer stage, especially infiltrative tumor growth and invasion, NKA-IFNγ might be related to the prognosis of GC patients. NK activity might also be useful as a biomarker to develop effective immune-stimulating drugs and new strategies for cancer treatment.

In conclusion, although further prospective longitudinal studies are required to explain the precise mechanism involved in decreased NKA in GC patients, our results suggest that NK cell activities for IFNγ production could be used as a supportive non-invasive tumor marker for GC.

## MATERIALS AND METHODS

### Patients

This study included 261 patients with newly diagnosed GC. They visited Seoul St. Mary’s Hospital Cancer Center from July 2015 to August 2016. They were diagnosed as GC based on histology study or endoscopic biopsy. None of these patients received preoperative treatment or chemotherapy. Their cancer stages were determined according to the 7th American Joint Committee on Cancer (AJCC) GC TNM Staging System [37, 38]. Medical records of patients were reviewed to obtain information for gender, age, laboratory findings, and pathologic features including tumor stage, pattern of tumor growth, invasion, tumor size, and metastasis. As control group, 48 healthy donors were also investigated. All participants were free from any inflammatory diseases without prior use of immunosuppressive agents. This study was approved by the Institutional Review Board of Seoul St. Mary’s hospital.

### NKA-IFNγ assasy

NKA-IFNγ was determined by enzyme immunoassay using NK Vue-Kit (ATgen, Sungnam, Korea) as described previously [8]. Fresh whole blood (1 ml) was obtained using tubes containing PromocaH and RPMI 1640 media and incubated at 37°C less than 1 hour after collection. After incubation at 37°C for 20-24 hours, cell-free supernatants were harvested and IFNγ levels were measured according to the manufacturer’s instructions. Briefly, 50 μL of six standards, controls, and samples were incubated in anti-human IFNγ coated plate at room temperature for 2 hours and washed with washing buffer (0.05% Tween 20 in PBS, pH 7.4). After the addition of IFNγ conjugate, the plate was incubated at room temperature for 1 hour and washed. After washing, 100 μL of substrate solution was added and incubated at room temperature for 30 min in the dark. Absorbance value was measured at wavelength of 450 nm. Concentrations of IFNγ were determined with a calibration curve. The measuring range was 0.1-4000 pg/mL and total imprecision for two levels of controls were less than 15% CVs.

### Routine tumor marker tests (CEA, CA19-9)

Serum CEA and CA19-9 levels were measured using ADVIA Centaur XP (Siemens Diagnostics, Tarrytown, NY, USA) and CA 19-9 ANTIGEN IRMA Kit (IMMUNOTECH, Pragu, Czech Republic). Normal reference levels for CEA and CA19-9 were ≤ 5.0 ng/mL and 36.1 U/mL, respectively.

### Statistical analysis

Statistical analyses were performed using MedCalc (v.16.4.3). Mann-Whitney U test and t test were used to compare unpaired two-group data. Performance characteristics of tests and combined tests for the diagnosis of GC were obtained using receiver operating characteristic (ROC) curve and multivariate logistic regression analysis. Results are presented as median and 95% confidence interval (CI) or mean ± standard deviation (SD). All *P* values were two-tailed. Statistical significance was considered at *P* < 0.05.
